# Seaweed as bioindicators of organic micropollutants polycyclic aromatic hydrocarbons (PAHs) and organochlorine pesticides (OCPs)

**DOI:** 10.1007/s11356-022-18634-z

**Published:** 2022-01-18

**Authors:** Gehan Mohamed El Zokm, Mona Mohamed Ismail, Mohamed Abd Elaziz Okbah

**Affiliations:** National Institute of Oceanography and Fisheries, NIOF, Cairo, Egypt

**Keywords:** Macroalgae, Mediterranean Sea, Hazard quotient, Pollutants, Risk value

## Abstract

This study highlights the role of seaweed as important indicators of pollutants as they respond immediately to change in water chemistry and have high survival capabilities. Concentration and risk assessment of 13 polycyclic aromatic hydrocarbons (PAHs), as well as 20 organochlorine pesticides (OCPs), were examined in the seaweed from El-Mex Bay, Mediterranean Sea during spring and autumn seasons. The green alga *Ulva compressa* had a maximum ability to accumulate both PAHs and OCPs. In general, the content of the tested micropollutants in the collected seaweed is correlated to their species, morphology, concentration, and nature of pollutant. Naphthalene (NAP) and benzo a pyrene were the predominant polycyclic aromatic hydrocarbons in all species with mean concentrations of 68.57 and 56.14 ng g^−1^, respectively. The results of the current study showed that the contribution of the different fractions of PAHs from the total concentration was as follows: fossil-fuel derived polycyclic aromatic hydrocarbons (∑PAH_F_; 49.32%) > combustion-derived polycyclic aromatic hydrocarbons (PAH_COMB_; 30.83%) > carcinogenic fractions (PAH_CARC_; 19.86%). A maximum PAH_CARC_ (30.38%) was recorded in *Ulva fasciata*. For OCPs, the presence of 1,1-dichloro-2, 2-bis (4-chlorophenyl) ethane (DDD) (ND-27.8 ng g^−1^) rather than DDT; 1,1,1-trichloro-2, 2-bis (4-chlorophenyl) ethane was an indication for biotransformation involving the reductive dichlorination of DDT to more recalcitrant and toxic DDD. Endrin ketone has the highest mean hazard quotient (0.376). The cancer risk values of most PAHs and OCPs were in the range from 10^−4^ to 10^−3^ recommending precautionary measures. The results explained that the present algal species play a vital role in the uptake of organic pollutants and act as biomarkers for micropollutants in the ecosystem.

**Supplementary Information:**

The online version contains supplementary material available at 10.1007/s11356-022-18634-z.

## Introduction

Marine ecosystems receive fluxes of organic and inorganic pollutants originating from natural and/or anthropogenic sources (Mohamed et al. 2016; Said et al. 2017; El Zokm et al. 2020a, b, c). Polycyclic aromatic hydrocarbons (PAHs) and organochlorine pesticides (OCPs) are toxic substances that can be transported over long distances via water or wind, and they tend to affect the environmental biota and human health in various ways (Olisah et al. 2020). The adverse effects of these compounds on the ecosystem are a major topic of discussion worldwide. Seaweeds have been identified as cost-effective natural agents able to capture different pollutants from the ecosystem owing to their ability to uptake contaminants into their tissues; as a consequence, they are considered good bioindicators of environmental pollution (Ismail and Ismail 2017; Saldarriaga-Hernandez et al. 2020). PAHs comprise two or more condensed aromatic rings. Their presence in the aquatic environment is assumed to be traceable using four sources: fuels (petrogenic), incomplete combustion processes (pyrogenic), organic metabolism (biogenic), and transformation processes taking place in sediments (diagenetic) (Honda and Suzuki 2020). Petrogenic sources are the major contributors of low-molecular-weight (LMW) PAHs (i.e., PAH comprising two or three condensed rings; molecular weight (MW) < 200 g/mol); by contrast, pyrogenic sources provide mainly high-molecular-weight (HMW) PAHs (i.e., PAHs comprising more than four rings; MW > 200 g/mol) (Marris et al. 2020). The toxicity of PAHs has to do with their carcinogenicity and non-biodegradable (Sun et al 2020); in fact, these compounds enter the cell as a result of their hydrophobic characteristics and prompt the expression of the genes encoding for members of the cytochrome P450 (CYP) enzyme group, which in turn activate PAHs to highly reactive diol-epoxides that combine with deoxyribonucleic acid (DNA) and become carcinogenic (Shimada and Fujii-Kuriyama 2004). Notably, LMW PAHs are acutely toxic but non-carcinogenic to most aquatic organisms; however, HMW PAHs are both carcinogenic and mutagenic (Karlsson and Viklander 2008). The International Agency for Research on Cancer named three PAHs as being probably carcinogenic (group 2A): dibenz[a,h]anthracene, benzo[*a*]pyrene (BaP), and benzo[*a*]anthracene (BaA) (Jacob 2008; Rengarajan et al. 2015). The US Environmental Protection Agency (EPA) has defined sixteen PAHs as seniority pollutants (Almatari et al. 2017).

The bioaccumulation of pesticide residues in seaweed has received increasing attention over the past few decades, and evidence has pointed to the effectiveness of seaweed species as biomonitors (Sundhar et al. 2019; Banach et al. 2020). Unfortunately, OCPs are a major group of cheap pesticides, and they are used in agriculture, public health, industry, and the household. Many studies are concerned with PAHs and pesticides in Egypt (El Deeb et al. 2007; Khairy et al. 2009; El-Naggar et al. 2018; Malhat et al. 2018). The chloride atoms Cl on the organic moieties of the OCPs render these compounds very stable in the environment. Studies have found a correlation between OCP exposure and various types of cancer, neurological damage, and endocrine system disruption **(**Malhat et al. 2018**).** Many OCPs are known or suspected to be hormone disruptors, and evidence from recently published studies indicates that exposure of the womb to extremely low levels of these compounds can cause irreversible damage to the human reproductive system (Malhat et al. 2018). The mode of action of pesticides is to inhibit acetylcholinesterase enzyme (AChE), leading to an accumulation of endogenous acetylcholine in the nervous system up to toxic levels, which in turn disturbs the system’s function (O’Brien 1967). Generally, in developing countries, pesticides should be used carefully, because toxicity outbreaks are often attributed to misuse of these substances. Green seaweed species like *Ulva rigida* and *Valonia utricularis* and red seaweed species like *Corallina elongate* and *Gracilaria cornea* have been used for OCP monitoring (Vega-Moreno et al. 2007). Notably, these species are also used in different economical applications, such as in human nutrition, animal food production, and beauty product manufacture (El Zokm et al. 2020a, b, c; Ismail et al. 2020). *U. rigida* and *G. cornea* are also reported to capture polychlorinated biphenyls (PCBs) and chlorinated pesticides, so these seaweed species can act as biomonitors of the said pollutants (Moy and Walday 1996; Pavoni et al. 2003). Consequently, these toxic compounds can enter the food chain, resulting in risks for the human health, through the consumption of edible seaweed and/or through use of seaweed as a natural fertilizer (Dhargalkar and Verlecar 2009). Importantly, PAHs have been known to affect a variety of biological processes and can be potent cell mutagens and carcinogens (Pelkonen and Nebert 1982). Persistent organic pollutants tend to accumulate in the food chain, so they may pose serious threats to higher trophic levels of aquatic communities and humans (Inguez et al. 2020). OCPs have been proven to have the potential to cause diabetes (Cox et al. 2007). Indeed, an impaired glucose metabolism has been proposed as a possible mechanism for the association between diabetes and OCP exposure, because OCPs are highly lipophilic and accumulate in human tissues. OCPs are highly chemically stable molecules, because they are constructed from C–C, C–H, and C–Cl bonds, which tend to be chemically inactive under normal environmental conditions. As a consequence, traces of organochlorine compounds can be found in the air and water throughout the world (Gad 2010; Malhat et al. 2018). Given that PAH and OCP pollution of the natural environment is a cause of grave concern worldwide due to these chemical species’ harmful effects on organisms, it is desirable to examine the residue levels of these organic micropollutants in food and assess the risk associated with exposure to these chemicals due to food consumption. El-Mex Bay, west of Alexandria city, is a Mediterranean coastal bay to the west of Western Harbor. This bay is exposed to large amounts of organic matter coming directly from industrial outlets (in particular the Alexandria Petroleum Company, the Alexandria Misr Petroleum Company, and the Misr Chemicals Industries Company) and indirectly from Lake Mariout via the El-Mex pump station. Importantly, El-Mex Bay has a relatively slow rate of self-purification (Said et al. 2017). Over time, OCPs and PAHs have been discharged in the El-Mex Bay from wastewater, agriculture drainage, chemical and electrical industries, and via atmospheric deposition.

Many strategies have been developed for mitigation of organic micropollutant (e.g., OCPs and PAHs) to reduce water pollution. The nanofibrous hypercrosslinked cyclodextrin networks (HCN) membrane can be utilized to remove organic micropollutants from ecosystems quickly and effectively (Topuz et al. 2021). In addition to chemical pretreatment, “ClO_2_ oxidation” is an effective method for removing PAH from soil via one-electron transfer, HOCl as a second oxidant, and ^•^OH participation (Sun et al. 2020). Nanocomposite hydrogels based on sustainable cellulose acetate are being used to remove various contaminants from both aqueous and organic media (Alammar et al. 2020). Shuang et al. (2017) detected the efficiency of washing technology for rapid remediation of soils or sediments by using a mixed surfactant of Triton X-100/SDS in different concentrations.

The aim of the present study was to detect the variation in the ability of seven seaweed species that grow in El-Mex Bay to uptake 13 PAHs and 20 OCPs, focusing on combining relationship indices, human risk indices, and carcinogenic risk assessment.

## Material and methods

### Collection of macroalgal species

**Fig. 1:**
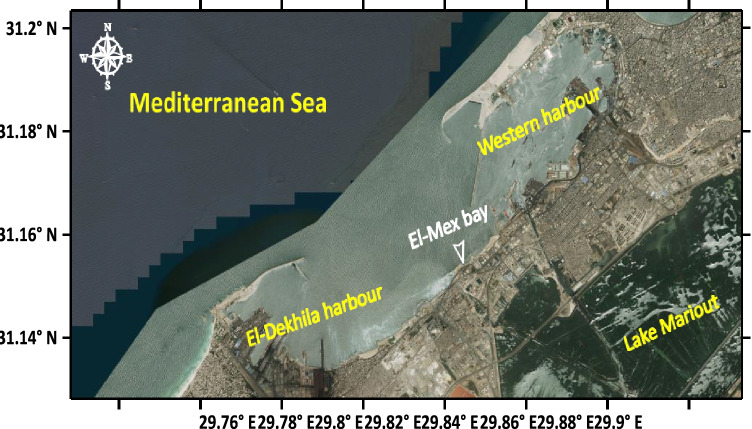
Map of El-Mex Bay, Alexandria coast, Egypt

Seaweed species were handpicked from rocks in the spring and autumn seasons of 2020 from El-Mex Bay, which is situated at longitude 29° 41.1′ to 29° 50.4′ E and latitude 31° 7.5′ to 31° 9′ N, Mediterranean coast, Alexandria, Egypt (Fig. 1). This area of the bay is a semi-closed marine basin that suffers from the discharges of different sources, such as agricultural waste, industrial wastes, and some oil contamination (Mohamed et al. 2016). The open connection between the bay and the Western Harbor of Alexandria facilitates the passage into the bay of petroleum contaminants released in association with ship traffic in the harbor. The newly established Dekheila harbor and the discharge of the waste of many petroleum companies of the petroleum complex through the Wadi Al-Kamar drain represent other sources of oil pollution in the bay.

**Fig. 2:**
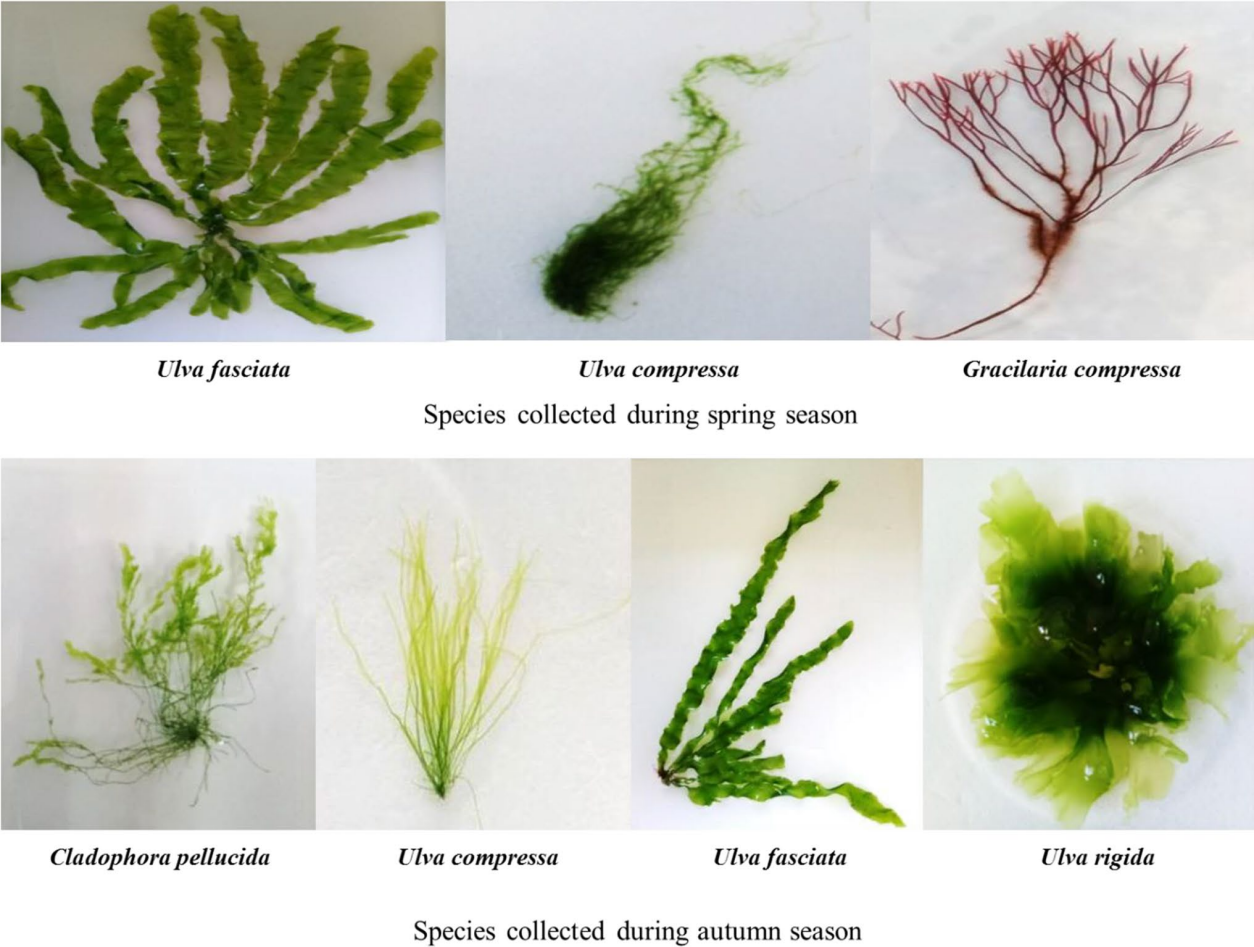
The collected seaweed from El-Mex bay

The collected samples were thoroughly washed in distilled water and cleaned using a soft brush to remove the deposits and epiphytes; subsequently, a portion of the fresh seaweed was processed as herbarium specimens. Other fresh samples were preserved in 5% formalin in seawater for taxonomical classification. A final portion was air-dried in the shade at room temperature then ground to obtain a fine powder, which was stored at − 20 °C until utilization. The taxonomy of the seaweed samples was carried out according to Aleem (1993), Jha et al. (2009), and Kanaan and Belous (2016). The names of the species were verified according to Guiry and Guiry (2020) and the Algae Base website. The collected species were identified as the green seaweed *Ulva compressa* Linnaeus (Uc1; Uc2) and *Ulva fasciata* Delile (Uf1; Uf2), which were collected during spring and autumn seasons, respectively. The red seaweed *Gracilaria compressa* (C. Agardh) Greville (Gc1) was harvested only during spring. On the other hand,* U. rigida* C. Agardh (Ur2) and *Cladophora pellucida* (Hudson) Kützing (Cp 2), which belong to the Chlorophyta, were sampled only during the autumn (Fig. 2).

### Reagents and standards

All the chemicals and solvents were purchased from Supelco (Bellefonte, Pennsylvania) and were chromatographic grade for pesticide residue analysis; the purified water was obtained from a Milli-Q water system (Millipore, Bedford, Massachusetts). OCP and PAH standards were also purchased from Supelco. Both standard stock solutions were dissolved in the suggested solvents with methanol/dichloromethane (1:1, v/v) for PAHs and n-hexane for OCPs and stored at 4 °C. The working solutions were prepared daily with suitable dilutions before use. NaCl, anhydrous Na2SO4, and Silica gel 60 were purchased from Merck, Germany. Water was purified with a Milli-Q System (Millipore, Bedford, MA, USA). All chemicals and reagents were of analytical grade and of the highest purity possible. Sigma-Aldrich (France) provides hexane and dichloromethane used for the extraction of OCPs. The florisil used in the cleaning was obtained from Merck (Germany). The International Atomic Energy Agency (IAEA) provides two external standard mixtures, the first containing (hexachlorobenzene, lindane, DDT, 1,1-dichloro-2,2-bis(4-chlorophenyl) ethylene (DDE), DDD) and the second standards mixture containing (heptachlor, aldrin, dieldrin, endrin) and a mixture of internal standards containing (PCB 29, PCB198, ɛ 1,2,3,4,5,6-hexachlorocyclohexane (HCH) and Endosulfane Id4).

### Sample treatment

Ten grams of each dried algal species was weighed exactly and homogenized with 30 g of anhydrous sodium sulfate in a mortar for 5 min. Ultrasonic extraction was then carried out on the homogenized samples using 200 mL of methanol for 2 h followed by 20 mL of 0.7 M potassium hydroxide, KOH, and 30 mL of distilled deionized water for 2 h. The extracts thus obtained were extracted three times with 80 mL of n-hexane in a 1000-mL separatory funnel. Thereafter, the extracts were concentrated to 1 mL under reduced pressure using a rotary evaporator (Kuderna-Danish); subsequently, 2 mL of cyclohexane was added to the concentrated extracts. The resulting solutions were fractionated and cleaned up by permeation chromatography (silica gel/alumina column). Elution was performed using a 25-mL mixture of n-hexane:methylene chloride (3:2 v/v). All eluates were concentrated to < 1 mL in volume, and 5 ml of acetonitrile was added to each eluate sample, which was then stored in a screw-cap high-performance liquid chromatography (HPLC) vial (Hein et al. 1988).

### Separation and quantification

The separation and quantification of PAHs and OCPs were carried out using a HPLC system (Agilent 1260 series). Prior to injection into the HPLC system, all solutions were degassed for 10 min in an ultrasonic bath. The HPLC system was calibrated using mixed standard solutions of PAHs and OCPs at five levels of concentration (10, 30, 60, 80, 100 ng L−1). All solutions were injected in triplicate to assure the repeatability of the procedure. The tested compounds were identified based on the correspondence of their retention times in the analyte samples with those of in standards. The injection volume was 20 μL for each sample solution. The column temperature was maintained at 35 °C.

### Estimated daily intake for an adult (EDI)

The estimated daily intake (EDI) was calculated with the following equation (Eq. (1); Health Consultation, Land Crab Evaluation, National Oceanographic Atmospheric Administration Data 2006)**:**

1 $$\mathrm{EDI }[\mathrm{mg}/\mathrm{kg}/\mathrm{day}] = [\mathrm{C }\times \mathrm{ IR }\times \mathrm{ EF }\times \mathrm{ ED}]/[\mathrm{BW }\times \mathrm{ AT}]$$

In Eq. (1), C is the residue concentration of the contaminant (ng g^−1^), IR is the ingestion rate (0.227 kg/day (8 oz. meal) for an adult), EF is the exposure frequency or the number of exposure events per year of exposure (365 days/year), ED is the exposure time (70 years), BW is the body weight (70 kg), and AT is the averaging time (non-cancer/lifetime = ED × 365 days/year).

2 $$\mathrm{Hazard quotient }(\mathrm{HQ}) =\mathrm{ EDI}/\mathrm{RFD}$$

In Eq. (2), RFD is the oral reference dose for non-carcinogenic effect (US EPA 2001).

The hazard index (HI) is the total mean concentration of pollutants present in seaweed. On the basis of the US EPA (2007) guidelines, an HI value below 1 indicates that no health risk is expected; by contrast, an HI value ≥ 1 is indicative of a moderate or high risk, suggesting adverse effects on human health. Specifically, the value of HI is calculated according to Eqs. (3) and (4):

3 $$\mathrm{HI }= \sum \left({\mathrm{HQ}}_{1} + {\mathrm{HQ}}_{2}+{\mathrm{HQ}}_{3}+ {\mathrm{HQ}}_{4} +\cdots {\mathrm{HQ}}_{\mathrm{n}}\right)$$

4 $$\mathrm{CRI }=\mathrm{ EDI }\times \mathrm{ CSF}$$

In Eq. (4), CRI is the cancer risk index and CSF is the cancer slope factor as described by the Integrated Risk:

5 $$\begin{array}{ccc}\mathrm{Information}& \mathrm{System}& \mathrm{RI}\end{array} (\mathrm{risk \ index}) = \sum \mathrm{ CRI}$$

### Quality control and assurance

In order to conduct the quality control experiments, a reference seaweed material (IAEA-140/OC; seaweed) was used for PAHs. Twenty standards sourced from Sigma-Aldrich (St. Louis) were analyzed for organochlorine pesticides (*n* = 3) together with the analyte samples. Analysis results for the reference materials for PAHs were within the range of certified values specified for the target compounds, with ranges of recoveries measured between 65 and 115%, except for the case of NAP (55.14%), a result that may be due to the high volatility of this compound, which renders it to accumulate in aqueous phases. Quality control standards were included throughout the sample analysis process, so as to monitor instrument performance. The instrument was calibrated by injecting the standard component mixture at five different concentrations, prior to sample analysis (Said et al. 2017). Recovery rates for samples spiked with OCP standards of 10, 20, 50,100, and 150 ng g^−1^ concentrations were measured in the 72–120% range. However, standard solution was used to verify the identification of each PCB congener with recoveries between 85 and 92% throughout all sample analyses. Analytical blanks in five replicates were included in all sample analyses. In order to increase the quality of the results, every four analyses, a blank experiment was conducted whereby a deionized water sample was analyzed implementing all the extraction procedures, which in turn afforded a clean background. Calibration curves were drawn using a set of standard solutions with definite concentrations of the analytes; these curves were characterized by good linearity (r > 0.99) and bias (2%). Furthermore, precision exhibited as relative standard deviation was agreed to be within 12%. Limits of detection (LOD) and of quantification (LOQ) are the most important values that researchers look for when considering method validity. The LOD and LOQ were determined using signal-to-noise ratio (SN), the experimental LODs were determined at a signal-to-noise ratio (S/N) of 3, and the limits of quantification (LOQs) were determined at S/N of 10 at the optimum λm and λx of each compound. The detection limits of individual compounds using the present method were determined as the concentration of analytes in a sample that yielded a peak signal-to-noise ratio (S/N) of 3, which were in the range of 0.01–0.20 ng g^−1^dry weight (dw) for PAHs and 0.001–0.004 ng g^−1^ dw for OCPs. The results of quantification (LOQ) were in the range of 0.04–0.60 ng g^−1^dw for PAHs and 0.005–0.017 ng g^−1^dw for OCPs.

### Statistical analysis

The statistical analysis for the assessment of the residue levels of PAHs and OCPs in the collected algal samples was carried out using Microsoft Excel 2010 and the Statistical Package for the Social Sciences; SPSS software version 22.0 for Windows.

## Results and discussion

### Levels of PAHs, relationship indices, and diagnostic ratios in the collected seaweed

**Fig. 3:**
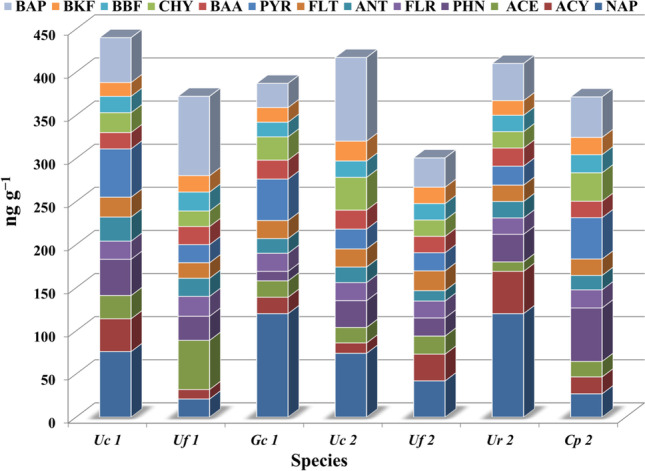
The residue levels of individual PAHs in the tested seaweed from El-Mex Bay Mediterranean Sea

The concentrations of 13 PAHs were determined in seven seaweed species, as can be evinced from Fig.3. In descending order, the level of PAH bioaccumulation in the seaweed samples was as follows: Uc1 > Uc2 > Ur2 > Gc1 > Cp2 > Uf1 > Uf2. Analysis results are indicative of large variations in PAH bioaccumulation among the seaweed species, with the measured concentration ranging from 300.6 ng g^−1^ in Uf2 1 to 440 ng g^−1^ in Uc1.

The difference in PAH concentration between algae samples may be related to algae species, morphology, physiology, and collection seasons. The level of pollutants in a particular algal species may depend greatly on the stage of growth and/or the condition of the fronds, given that old, decaying fronds can contain much greater amounts of pollutants (Maroli et al. 1993). In addition, seaweed sensitivity to chemical pollutants varies between species and their lipid content (Yunker et al. 2002). The hydrophobicity and lipophilicity degree of hydrocarbons might facilitate or hinder the accumulation process inside algal cell (Erickson 1997). Moreover, the thallus of *U. compressa* is more branched than that of *U. fasciata*, so the receptor sites on the thallus surface and uptake rate increased. Notably, *Ulva* and *Enteromorpha* species are generally used as bioindicators of contamination (Ismail and Ismail 2017; El-Shoubaky and Mohammad 2016).

The values for the mean residue levels (ng g^−1^) of PAHs in the tested species were observed to be in the following descending order: NAP (68.57) > BaP (56.14) > pyrene PYR (34.14) > phenanthrene (PHN) (32.43) > acenaphthylene (ACY) (25.66) ≈ chrysene (CHY) (25.29) ≈ acenaphthene (ACE) (24.43) > fluorene (FLR) (20.80) ≈ fluoranthene (FLT) (20.57) ≈ BaA (20.43) ≈ benzo (b)fluoranthene (BBF) (19.43) ≈ anthracene (ANT) (18.81) > benzo (k)fluoranthene (BKF) (18.71). In general, the rapid biotransformation of some PAHs by seaweed may lead to their disappearance or to a decrease in their concentrations (Pavoni et al. 2003).

Analysis results indicated that the total concentration of fossil fuel-derived PAHs (∑PAH_F_; (PHN + ANT + FL + ACE + ACY + NAP) was in the range (146.60–250.00 ng g^−1^). The total concentration of the combustion-derived PAHs (∑PAH_COMB_; (FLT + PYR + BKF + CHY + BaA + BaP + BBF)) ranged from 76.00 to 120 ng g^−1^, while the total concentration of the carcinogenic PAH fractions ∑PAH_CARC_; BaA + BaP varied between 67.00 and 135.00 ng g^−1^. The relative percentages and the ratios of PAH fractions are listed in Table 1. Notably, the value of the ∑PAH_F_ indicated that fossil fuel-derived PAHs provided the highest contribution to the PAHs, making up 49.32% of the total; moreover, combustion-derived PAHs made up 30.83% of the total PAHs, and carcinogenic PAHs made up 19.86% of the total PAHs. The Joint Food and Agriculture Organization and the World Health Organization Expert Committee on Food Additives consider BaP level as a general carcinogenic indicator of ∑PAHs and recommended 10 ng g^−1^ as the tolerance limit for the concentration of the said species (Lawal 2017). In the present study, the detected total level of BaP ranged from 28 to 92 ng g^−1^, a concentration range that is much higher than the mentioned tolerance limit.

**Table 1:** Relationship indices of PAHs in the collected seaweed species from El-Mex Bay

Relationship indices	Spring			Autumn			
	***Uf1***	***Uc1***	***Gc1***	***Ur2***	***Uc2***	***Uf2***	***Cp2***
**PHN/ANT**	1.33	1.5	0.65	1.68	1.72	1.75	3.71
**ANT/(ANT + PHN)**	0.43	0.4	0.61	0.37	0.37	0.36	0.21
**BAA/CHY**	1.17	0.83	0.81	1.11	0.58	1	0.58
**BBF/BAP**	0.24	0.37	0.61	0.44	0.2	0.56	0.45
**FLR/(FLR + PYR)**	0.52	0.27	0.3	0.46	0.48	0.48	0.3
**FLT/PYR**	0.86	0.41	0.44	0.86	0.91	1.1	0.4
**BAP/ BAP + CHY**	0.836	0.693	0.509	0.694	0.719	0.642	0.588
**BAA/(BAA + CHY)**	0.54	0.45	0.45	0.53	0.37	0.5	0.37
**∑PAHF**	161	232	207	250	174	146.6	164.3
**∑PAHCARC**	135	90	67	83	138	72	87
**∑PAHCOMB**	76	118	113	77	105	82	120
**%∑PAHF**	43.28	52.73	53.49	60.98	41.73	48.77	44.25
**%∑PAHCARC**	36.29	20.45	17.31	20.24	33.09	23.95	23.43
**%∑PAHCOMB**	20.43	26.82	29.2	18.78	25.18	27.28	32.32
**LPAHs / HPAHs**	0.763	1.115	1.15	1.563	0.716	0.952	0.763

Diagnostic fingerprint ratios of residue levels for various PAHs in seaweed species were calculated, as can be evinced from Table 1; these data are important for identifying and estimating the contribution of potential pollution sources (Kucuksezgin et al. 2013). In the current study, PHN/ANT showed less than 10 that all algal samples were affected by combustion residues (Yunker et al. 2002). A value for the FLA/PYR concentration ratio over 1 indicates a pyrolytic origin for the PAHs, and the values for this ratio in the present study ranged from 0.4 to 0.91, indicating the prevalent origin of PAHs to be fuel oil and crude oil combustion, with a small contribution from wood combustion only over species with a value of 1.1 (Gschwend and Hites 1981). A value for the ANT/(ANT/ + PHN) ratio above 0.10 is indicative of the dominance of combustion sources for PAHs (Yunker et al*.* 2001), and in the present study the said ratio ranged in value from 0.21 to 0.61. FLR/ (FLR + PYR) < 1 in all studied species may indicate the pyrolytic input. The BaA/(BaA + CHY) ratio was observed to range from 0.37 to 0.54, which, according to the results of previously published studies, is indicative of PAHs originating from both gasoline and diesel engines (Sicre et al. 1987). The BaP/ (BaP + CHY) ratio ranged from 0.51 to 0.84, which confirms the contribution of both diesel and gasoline emissions to PAH contamination (Guo et al. 2003). The BBF/BaP ratio in most seaweed species was observed to have values below 0.5, indicating the origin of PAH contamination to be pyrolytic. In the current study, the BaA/CHY ratio was characterized by values in the 0.58–1.17 range. Yunker et al. (2002) suggested that a value for the BaA/CHY ratio ≥ 0.5 is indicative of PAHs originating from vehicle emissions. Finally, the LMW PAH/HMW PAH ratio in the current study ranged between 0.76 and 1.56, well within the 0.6–2.3 range reported for PAHs derived from pyrogenics, especially the combustion of coal, diesel fuel, and coal tars (Stogiannidis and Laane 2015).

### Levels of OCPs in the seaweed species analyzed

**Fig. 4:**
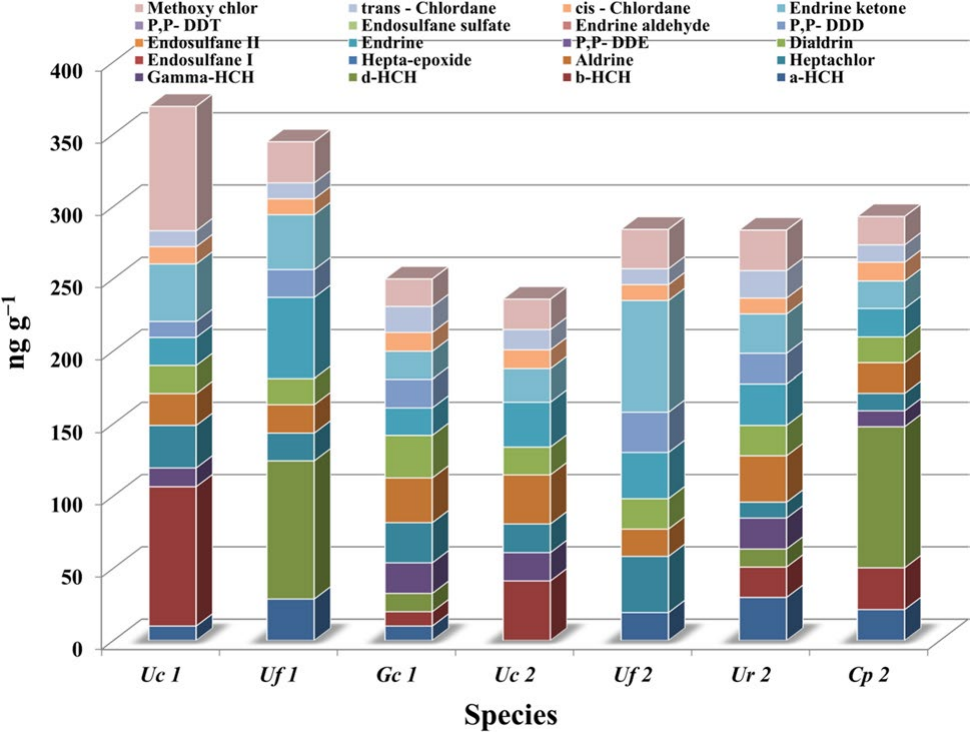
The residue levels of OCPs in the tested seaweed from El-Mex Bay Mediterranean Sea

Regarding the total OCPs, the order in decreasing OCP concentration in the collected seaweed samples was the following: Uc1 > Uf1 > Cp2 > Uf2 > Ur2 > GC1 > Uc2 (Fig.4). The variability in the level of pesticide uptake by seaweed has been reported to be related to the biochemical composition of the algae, especially its lipid content, the affinity toward OCPs, the fruiting season, and other physical and chemical conditions of the marine environment (Sundhar et al. 2020). Notably, the biological sensitivity of seaweed to chemical pollutants is specific to the species (Lytle and Lytle 2001). Spring-collected samples of both *Ulva* species taken into consideration in the present study contained a high level of OCPs, an observation that may be related to these seaweed species’ leaf-shaped thallus, which is characterized by a high surface/volume ratio, as well as the season of collection (Pavoni et al. 2003).

Aldrin is an organochlorine insecticide that is dangerous and carcinogenic to humans, even at a low concentration of 25 mg m^−3^. This compound is also easily absorbed by seaweed. Complete depletion of heptaepoxide, Endosulfan 1, P,P-DDE, Endosulfan II, Endrin aldehyde, Endosulfan sulfate, P,P-DDT was observed in all the studied seaweed samples. In fact, due to their toxicity, endosulfan, methoxychlor, and DDT have been banned since 2012 in over 80 countries (US EPA 2006a, 2006b).

A degree of contamination among algal species was observed relative to the other examined pesticides (13 compounds). DDT undergoes a metabolic process whereby it is transformed into DDD. The presence of DDD (ND-27.8 ng g^−1^) rather than DDT was an indication of the occurrence of a biotransformation involving the reductive dichlorination of DDT and formation of DDD, which is regarded as a ubiquitous environmental contaminant and is often reported to be more recalcitrant and toxic than DDT.

High concentrations (ng g^−1^) were detected for d-HCH (ND-97.6), b-HCH (ND-96.4), endrin ketone (19.2–77), and methoxychlor (19–86). Generally, HCHs are reasonably stable compounds, which decompose to yield trichlorobenzene only under alkaline conditions. Furthermore, the accumulation of HCH in Cp2 (97.6 ng g^−1^ and UF1 (95.6 ng g^−1^) makes these seaweed samples ideal biomarkers for d-HCH contamination. On the other hand, the maximum concentrations of a-HCH (96.4 ng g^−1^) and methoxychlor (86 ng g^−1^) were observed in Uc1, so this seaweed sample is recommended as a biomarker for the mentioned contaminants. Notably, substantially higher amounts of endrin were observed to accumulate in Uf1 than in the other tested seaweed species, due to *U. fasciata* being characterized by higher lipid contents than *U. compressa* and other seaweed species, as documented by Ismail et al. (2017) and El Zokm et al. (2020c). Evidence from a previously published study indicated that endrin accumulates in the fatty tissues of living organisms (UNEP 1995). The discovery of endrin ketone in all the seaweed samples analyzed herein is reflective of the continued uses of endrin in this region; importantly, the half-life of this compound can have a value of up to 12 years in the soil, and thus endrin ketone remains in the ecosystem for decades (UNEP 1995). As can be evinced from the reported data, the seaweed samples collected during the spring displayed higher OCP concentrations (mean concentration: 321.23 ng g^−1^ than those collected during the autumn (mean concentration: 274.12 ng g^−1^) confirming the important effect that sampling time has in the present context. These differences are associated with algal metabolic activity and fruiting time; indeed, most types of seaweed grow faster and display maximum productivity during the spring (Wilde, 2017). Notably, Sundhar et al. (2019) reported that bioaccumulation of HCH residues in seaweed was found to be associated with the fruiting season of the specific seaweed. In general, the ability of seaweed to bioaccumulate OCPs varies according to seaweed morphology (e.g., surface size and characteristics as well as texture), chemical composition (especially lipid content), and life cycle (permanent, seasonal), as well as according to the chemical composition and solubility of the pollutants (Yunker et al. 2001; Pavoni et al. 2003).

### Human risk assessment

The slower the rate of decomposition of a toxic substance the greater the risk of chronic poisoning, even if the environmental levels of the particular toxin are not very high. Notably, the RFD is a conservatively chosen threshold dose for the intake of a particular contaminant. Hence, if the estimated intake is less than the reference dose (HQ < 1), almost no possibility of an adverse health effect exists. By contrast, the same inference cannot be made if the said intake exceeds the RFD threshold (HQ > 1); as can be evinced from the data listed in Table 2, the HQ value for all studied pollutants was < 1.

**Table 2:** Calculated average estimated daily intake (DI) mg/kg/day, HQ hazard quotient and CRI cancer risk index of PAHs and OCPs

Organic contaminants		RfD	CSF	DI	HQ	CRI
NAP	**Poly aromatic hydrocarbons**	0.02	NA	9.54E − 04	1.11E − 02	NA
ACY		0.02	NA	1.50E − 04	4.16E − 03	NA
ACE		0.06	NA	8.34E − 05	1.32E − 03	NA
PHN		0.04	NA	2.22E − 04	2.63E − 03	NA
FLR		0.02	NA	8.32E − 05	3.37E − 03	NA
ANT		0.3	NA	7.92E − 05	2.03E − 04	NA
FLT		0.04	NA	1.05E − 04	1.67E − 03	NA
PYR		0.03	NA	6.75E − 05	3.69E − 03	NA
BAA		NA	0.73	6.10E − 05	NA	4.84E − 05
CHY		NA	0.007	6.67E − 05	NA	5.74E − 07
BBF		NA	0.73	1.11E − 04	NA	4.60E − 05
BKF		NA	0.073	6.62E − 05	NA	4.43E − 06
BAP		NA	7.3	8.20E − 05	NA	1.33E − 03
a-HCH	**Organochlorine pesticides**	0.8	6.3	6.44E − 05	6.90E − 05	8.38E − 05
b-HCH		0.8	1.8	1.07E − 04	1.14E − 04	3.81E − 09
d-HCH		NA	NA	1.77E − 04	NA	NA
Gamma-HCH		0.3	NA	5.58E − 05	1.33E − 04	NA
Heptachlor		0.5	4.5	7.32E − 05	1.46E − 04	3.30E − 04
Aldrine		0.03	NA	8.27E − 05	2.76E − 03	NA
Hepta-epoxide		0.013	9.1	ND	ND	ND
Endosulfane I		0.006	NA	ND	ND	ND
Dialdrin		0.05	16	6.75E − 05	1.35E − 03	1.08E − 03
*P,P-*DDE		0.5	0.34	ND	ND	ND
Endrine		0.3	NA	9.53E − 05	3.18E − 04	1.10E − 05
Endosulfane II		0.004	NA	ND	ND	ND
*P,P-*DDD		0.5	0.24	4.57E − 05	9.14E − 05	1.36E − 05
Endrine aldehyde		0.0003	NA	ND	ND	ND
Endosulfane sulfate		0.00005	NA	ND	ND	ND
*P,P-*DDT		0.5	0.34	ND	ND	ND
Endrine ketone		0.0003	NA	1.13E − 04	3.76E − 01	NA
*cis*-Chlordane		0.0006	0.35	3.89E − 05	6.49E − 02	1.56E − 05
*trans*-Chlordane		0.0006	0.35	4.45E − 05	7.41E − 02	1.53E − 03
Methoxychlor		0.005	NA	1.06E − 04	2.12E − 02	NA

### Carcinogenic risk assessment

The carcinogenic risks associated with the detected pollutants were determined by estimating the EDI (mg/kg/day), incremental lifetime cancer risk (ILCR), and RI in macroalgae species. The ILCR and RI values for the carcinogenic PAHs and OCPs were calculated via Eqs. (4) and (5), respectively, according to the guidelines of the US EPA (Table 2) (Jamhari et al. 2014; US EPA 1989, 2015). In Eq. (4), CSF is the carcinogenic regression factor for an individual congener (US EPA 2015). The regression factors for both PAHs and OCPs are listed in Table 2. Pesticides can affect a variety of cancers through the immune mechanism. OCPs are neurotoxic pesticides that interfere with the function of the neurotransmitter γ-aminobutyric acid (GABA) and affect the human nervous system, liver, and kidneys (US EPA 2006a, 2006b). The risks associated with dietary exposure to individual PAHs were evaluated using the DI and HQ approach (Table 2). In particular, the fact that the DI values were < 10^−4^ indicates that the residue levels of PAHs in algal species in El-Mex Bay do not pose a significant health risk to consumers. The fact, furthermore, that the HQ values were < 1 for all analyzed seaweed samples, for both PAHs and OCPs, indicates that there is virtually no potential for adverse health effects. Nevertheless, for the initial quantitative assessment of the risk associated with OCP contamination, the threshold level is considered HQ ≤ 0.2; at HQ > 0.2, therefore, a further detailed risk assessment should be performed (Health Canada 2004). Accordingly, for endrin ketones for which a HQ value of 0.376 was calculated, an in-depth risk evaluation should be considered. The HI calculated in this study is very low (Fig. 4), indicating that no adverse effects are likely to arise. In addition, risk management decisions often depend on a range of cancer risks. The US EPA indicates a risk level of 10^−6^ as the threshold below which the carcinogenic risk is minimal; above this level, management decisions can be considered. In the case, however, of HI values equal to or above 10^−3^, preventive measures are required (US EPA 1996, 2011; Domingo and Nadal 2015). In the present study, the estimated cancer risk values calculated for most PAHs and OCPs ranged from 10^−4^ to 10^−3^, so precautionary measures are required.

## Conclusion

Contaminant monitoring is a critical component of the efforts to minimize the potential risks to human health posed by food pollutants. Seaweed are interesting model aquatic organisms to assess the impact of pollution on an ecosystem. According to the results of the present study, seaweed species belonging to the Ulvaceae family accumulated more pollutants than other seaweed species, indicating that specific accumulation activity of these seaweeds depends on their morphology, including branching, size, and nature of the blades. This algal family is characterized by survival in low and high-pollution areas throughout the year, so it can be recommended as a medium-term environmental pollution monitor. PAH bioaccumulated in seaweeds with concentrations varied from 300.6 ng g^−1^ in Uf2 1 to 440 ng g^−1^ in Uc1. Based on diagnostic fingerprint ratios, LMW PAH/HMW PAH (0.76 to 1.56) and ANT/(ANT/ + PHN) > 0.10, indicating that fuel combustion is their source. OCPS, on the other hand, revealed high concentrations (ng g^−1^) of d-HCH (ND-97.6), b-HCH (ND-96.4), endrin ketone (19.2-77), and methoxychlor (19-86). It is noteworthy that OCPs concentrations were greater in the spring (average: 321.23 ng g^−1^) than in the autumn (average: 274.12 ng g^−1^), confirming the importance of sampling time. The calculated daily intake of PAHs and OCPs <10^−4^ (mg/kg/day) did not reveal significant health risks to consumers. The hazard quotient does not exceed one in all the studied seaweed, and the hazard index was very low, so there is a possibility of non-carcinogenic effects. The cancer risk index of all studied algae for most PAHs and OCPs was less than 10^−3^. More work is needed to better understand the potential impacts and risks associated with exposure to PAHs and OCPs in trophic high-level organisms.

## Supplementary Information

Below is the link to the electronic supplementary material.

Supplementary file1 (DOCX 20 KB)

## Data Availability

All data generated or analyzed during this study are included in this published article.
