# Supplementary material for: Seaweed as bioindicators of organic micropollutants polycyclic aromatic hydrocarbons (PAHs) and organochlorine pesticides (OCPs)
Source: Environ Sci Pollut Res Int. 2022 Jan 18;29(23):34738–48. doi: 10.1007/s11356-022-18634-z (PMC9076741; doi:10.1007/s11356-022-18634-z)
Supplement: Supplementary file 1 — Supplementary file1 (DOCX 20 KB) [file 11356_2022_18634_MOESM1_ESM.docx]

| Data of Figure 4.The residue levels of individual PAHs in the tested seaweed from El-Mex Bay Mediterranean | | | | | | | |
| --- | --- | --- | --- | --- | --- | --- | --- |
|  | ***Uc* 1** | ***Uf* 1** | ***Gc* 1** | ***Uc* 2** | ***Uf* 2** | ***Ur* 2** | ***Cp* 2** |
| **NAP** | 76 | 21 | 120 | 74 | 42 | 120 | 27 |
| **ACY** | 38 | 11 | 19 | 12 | 31 | 49 | 20 |
| **ACE** | 27 | 57 | 19 | 18 | 21 | 11 | 18 |
| **PHN** | 42 | 28 | 11 | 31 | 21 | 32 | 62 |
| **FLR** | 21 | 23 | 21 | 21 | 19.6 | 19 | 21 |
| **ANT** | 28 | 21 | 17 | 18 | 12 | 19 | 18 |
| **FLT** | 23 | 18 | 21 | 21 | 23 | 19 | 19 |
| **PYR** | 56 | 21 | 48 | 23 | 21 | 22 | 48 |
| **BAA** | 19 | 21 | 22 | 22 | 19 | 21 | 19 |
| **CHY** | 23 | 18 | 27 | 38 | 19 | 19 | 33 |
| **BBF** | 19 | 22 | 17 | 19 | 19 | 19 | 21 |
| **BKF** | 16 | 19 | 17 | 23 | 19 | 17 | 21 |
| **BAP** | 52 | 92 | 28 | 97 | 34 | 43 | 47 |

| Data of Figure 3. The residue levels of OCPs in the tested seaweed from El-Mex Bay Mediterranean Sea | | | | | | | | | | | | | | | | | | | | |
| --- | --- | --- | --- | --- | --- | --- | --- | --- | --- | --- | --- | --- | --- | --- | --- | --- | --- | --- | --- | --- |
|  | a-HCH | b-HCH | d-HCH | Gamma-HCH | Heptachlor | Aldrine | Hepta-epoxide | E0osulfan I | Dialdrin | *P,P-* DDE | *P,P-* DDD | *P,P-* DDT | E0rin | E0rin aldehyde | E0rin ketone | E0osulfane II | E0osulfane sulfate | *cis* - Chlordane | *trans* - Chlordane | Methoxy chlor |
| *Uc* 1 | 10 | 96.4 | 0 | 13 | 29.4 | 22 | 0 | 0 | 19.2 | 0 | 11 | 0 | 19.4 | 0 | 39.8 | 0 | 0 | 12 | 11 | 86 |
| *Uf* 1 | 28.7 | 0 | 95.6 | 0 | 19.2 | 19.6 | 0 | 0 | 18 | 0 | 19.2 | 0 | 56 | 0 | 38 | 0 | 0 | 11 | 11 | 28.4 |
| *Gc* 1 | 10 | 9.9 | 12.7 | 21.1 | 27.8 | 31 | 0 | 0 | 29.4 | 0 | 19.7 | 0 | 19 | 0 | 19.2 | 0 | 0 | 13 | 18 | 19 |
| *Uc* 2 | 0 | 41.2 | 0 | 19.6 | 19.8 | 34 | 0 | 0 | 19.2 | 0 | 0 | 0 | 31 | 0 | 23 | 0 | 0 | 13 | 14 | 21 |
| *Uf* 2 | 19.3 | 0 | 0 | 0 | 38.9 | 18.9 | 0 | 0 | 21 | 0 | 27.8 | 0 | 32 | 0 | 77 | 0 | 0 | 11 | 11 | 27.2 |
| *Ur* 2 | 29.8 | 21 | 12.5 | 21.4 | 11 | 32.1 | 0 | 0 | 21 | 0 | 21 | 0 | 28.6 | 0 | 27.2 | 0 | 0 | 11 | 19 | 28 |
| *Cp* 2 | 21.4 | 28.9 | 97.6 | 11 | 12 | 21 | 0 | 0 | 17.8 | 0 | 0 | 0 | 19.7 | 0 | 19 | 0 | 0 | 13 | 12 | 19.6 |
